# The progressive model of perioperative care

**DOI:** 10.3389/fmed.2024.1398167

**Published:** 2024-09-27

**Authors:** Brandon Stretton, Aashray K. Gupta, Sanjana Santhosh, Stephen Bacchi, Joshua G. Kovoor

**Affiliations:** ^1^Adelaide Medical School, University of Adelaide, Adelaide, SA, Australia; ^2^Health and Information, Adelaide, SA, Australia; ^3^The University of Queensland Medical School, University of Queensland, Brisbane, QLD, Australia; ^4^Department of Surgery, Ballarat Base Hospital, Grampians Health, Ballarat, VIC, Australia

**Keywords:** perioperative medicine, hospital system, patient recovery, healthcare delivery, change management, resource modeling

## Introduction

Advancements in surgical and anesthetic techniques have permitted the successful completion of higher risk operations, with a simultaneous decline in intraoperative mortality. However, the global burden of postoperative mortality remains significant, particularly among severely ill patients undergoing emergency, pediatric, or cardiac surgeries (1). While these patients' underlying conditions are often the primary contributors to postoperative mortality, postoperative death (i.e., death from complications after surgery, not a direct result of the surgery itself) within 30 days of surgery, is now the third lead cause of death globally (2). Currently, the average patient requires several operations during their lifespan (3), and this will likely increase as the population above age 60 in most nations is expected to double by 2050 (approaching ~22%), half of whom will require surgery in their remaining life (4, 5).

## The need for a new model of perioperative care

Rates of postoperative death are a measure of the surgical care systems success (2), so whilst more targeted perioperative optimization and management strategies are required, so too are system-level changes. There is a growing recognition that optimizing perioperative care can play a crucial role in improving outcomes, more specifically, a new, *progressive*, system of inpatient care which is more accommodating to a patient's progress through their perioperative journey is required.

## Key components of the “progressive” model

The term “*progressive”* is deliberately chosen to convey both the advancement in perioperative care delivery that this model represents, as well as its alignment with the patient's *progressive* recovery journey post-surgery. The model is designed to be adaptable to the patient's changing needs throughout their recovery journey.

### Non-linear facility design: creating a flexible physical layout that allows for efficient patient flow and multidisciplinary collaboration

The *progressive* Model of Perioperative Care is designed to accommodate the dynamic and often unpredictable nature of a patient's perioperative journey by providing a flexible and responsive system of care. This model integrates a *progressive* approach where patients can adjust their resource requirements based on clinical acuity, ensuring that care is tailored to their immediate needs. The model emphasizes a non-linear topographical configuration of healthcare facilities, promoting efficient patient flow, multidisciplinary collaboration, and optimal access to necessary services. By rethinking the layout design, staffing strategies, and placement of medical equipment, this model seeks to enhance perioperative outcomes across all surgical disciplines. This approach is particularly timely given the global increase in surgical procedures and the growing elderly population, which is expected to double by 2050, with half of individuals over 60 likely to require surgery in their remaining lifetime (4, 5).

### Shared care model: involving multiple healthcare professionals, including surgeons, intensivists, anesthesiologists, and internists, in the patient's care

An individual patient's perioperative journey is not archetypal or pre-determined and needs to occur in within an individual system that can inherently accommodate unexpected alterations in the recovery journey. Despite preoperative risk mitigation, perioperative progress may still be dynamic and unpredictable. Accordingly, the perioperative system needs to be acquiescent to a patient's progress, with the ability to rapidly titrate clinical care as required. Such a *progressive* system requires a foundational consideration for facility topographical configuration, patient flow promotion, and encouragement of multi-disciplinary, integrated, patient-centric service delivery. Additionally, this *progressive* system must be conscientiously staffed, be equitably employable, adaptable and capable of evolution, particularly for novel technology.

**Figure 1:**
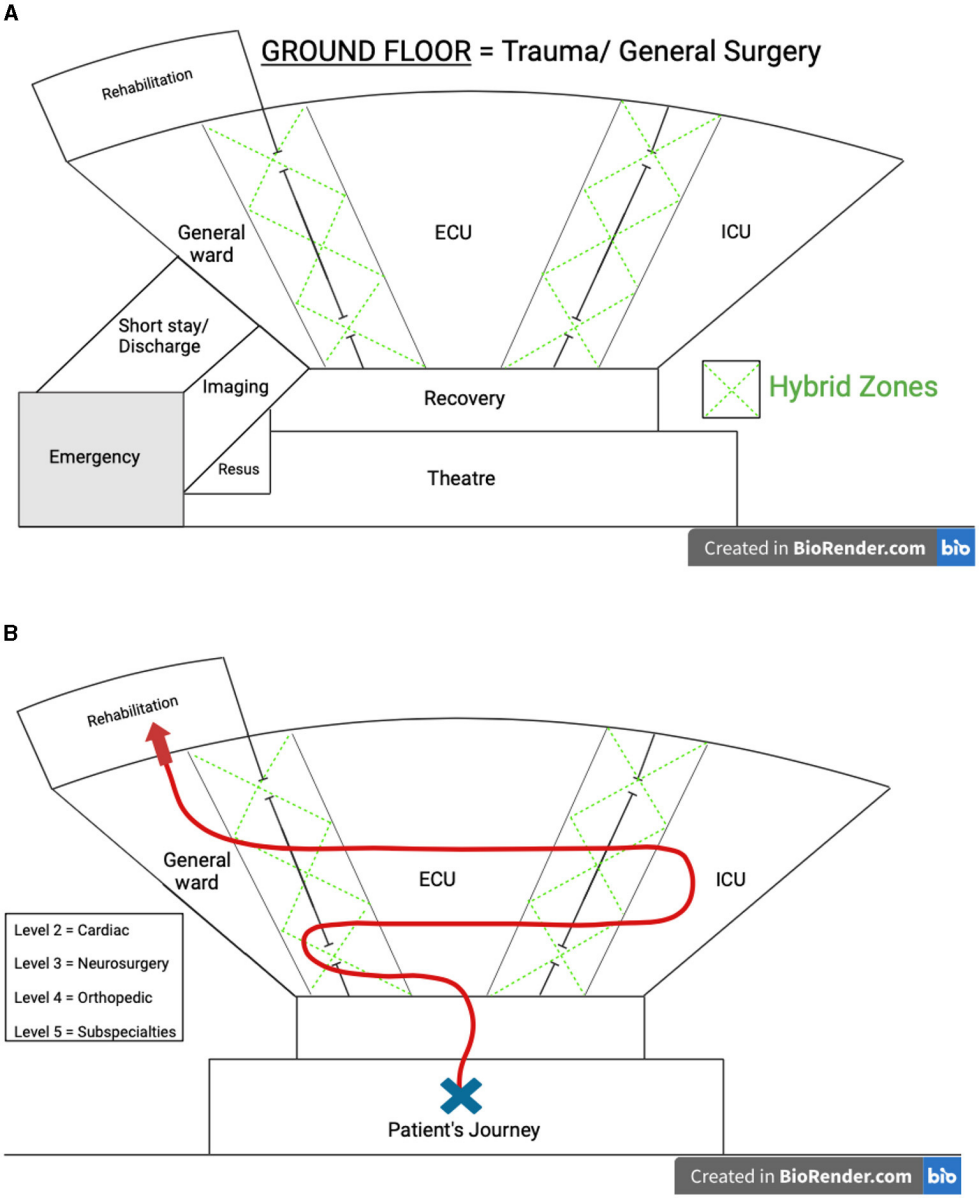
**(A)** Progressive perioperative care topographical model. **(B)** Patient journey through the progressive perioperative care model. The figure provided in our manuscript is intended to serve as a pictorial representation, emphasizing the importance of a non-linear topographical configuration and efficient patient flow within the proposed model. We acknowledge that the specific layout shown may not be directly applicable to every hospital, particularly those with existing architectural constraints. However, the core principle of the model—facilitating optimal patient flow and resource utilization—can still be implemented in more traditional ward designs. By intentionally organizing flow streams and processes, hospitals of various configurations can adopt this model to enhance perioperative care.

In their surgical care journey, patients will invariably encounter the theater, and recovery rooms. Thereafter, the patient's clinical condition may require the intensive care unit (ICU), enhanced postoperative care units (EPC) or a general ward before discharge. This progress is not linear, and so the *progressive* perioperative model necessitates these units demonstrate a non-linear physical proximity, to accommodate and optimally manage a patients' progress through their journey (Figure 1). Such a topographical layout allows for prompt patient flow, but also optimal access to, and prompt multidisciplinary consultation.

The *progressive* perioperative model supposes an alternative to the existing, “ward/home team” model, which does not standardize the proposed degree of integration (6), in place of a shared, integrated patient centric model of care which is waypoint orientated. A common denominator is required throughout this shared-care model, to provide context to a patient's current clinical condition and propose patient specific, appropriate investigations and interventions. To this end, surgeons will invariably be intimately involved throughout the patient's progress, however, throughout the postoperative phase, surgeons are better serviced by providing a consulting service, with expert focus on issues relating to the operation itself (the surgeon in fact knows the procedure, is aware of the complications and can spot when things are not right). Instead, “bed card/home team/primary care provider” is determined by a patient's residence in either ICU (intensivists), ECU (anesthetists) or general ward (physicians). The diversified skillset of internists (internal medicine specialists/general physicians) is integral to this model by providing this longitudinal service, and the diverse pathophysiological knowledge to escalate clinical care as required.

### Appropriate staffing: ensuring adequate staffing levels and skill mix for optimal patient outcomes

Appropriate staffing and defined clinical processes are required to deliver optimal care. Missed nursing care, which may be product of understaffing or inappropriate training may increase inpatient mortality upwards of 10% (3). The *progressive* model of perioperative care emphasizes the appropriate numbers and skill-mix of staffing. Each unit requires specialized nursing staff however, this model also affords opportunities for mutual skill development by process of transitioning care in the hybrid zones. Similar case mix and skill acquisition advantages can be shared with other allied health specialties integral to perioperative care such as physiotherapy, occupational therapists, podiatry and speech therapists.

### Adaptability: designing a model that can be implemented in various healthcare settings, including low- and middle-income countries

The *progressive* principle of perioperative care management discussed is designed to be adaptable and implementable across various healthcare settings, irrespective of the available resources. The model is structured to provide a flexible framework that institutions worldwide, regardless of their resource levels, can modify to suit their specific needs. Half of the postoperative deaths occur within Low-Middle income countries (LMIC) (7). Additionally, appropriate expansion of surgical service provision to address currently unmet needs would nearly double the rates of postoperative mortality in LMIC (7). Therefore, system changing models of care delivery must be amenable to implementation in this setting. The *progressive* perioperative model principles suggests utilization for optimal delivery of existing infrastructure, rather than requiring additional resources that could deplete systems and risk compromising patient care. This approach can be retrofitted into existing healthcare systems and implemented into LMIC without extenuating fiscal burdens.

## Potential benefits of the progressive model

By adopting this model, healthcare systems can improve patient outcomes, reduce postoperative mortality rates, and better prepare for the increasing demand for surgical care.

Ultimately, perioperative care requires more attention. As current health service delivery is ill-equipped to accommodate the impending increase in case numbers globally that accompanies a growing and aging population, the burden of postoperative deaths will continue to increase. The proposed system, a *progressive* perioperative care model is one such model that progresses the delivery of perioperative care in anticipation for the needs of future patients. This model will continue to progress and evolve in accordance with local demographic needs.
